# Multilocus Sequence Typing (MLST) for Lineage Assignment and High Resolution Diversity Studies in *Trypanosoma cruzi*

**DOI:** 10.1371/journal.pntd.0001049

**Published:** 2011-06-21

**Authors:** Matthew Yeo, Isabel L. Mauricio, Louisa A. Messenger, Michael D. Lewis, Martin S. Llewellyn, Nidia Acosta, Tapan Bhattacharyya, Patricio Diosque, Hernan J. Carrasco, Michael A. Miles

**Affiliations:** 1 London School of Hygiene and Tropical Medicine, London, United Kingdom; 2 Instituto de Higiene e Medicina Tropical, Lisboa, Portugal; 3 Departamento de Medicina Tropical, Instituto de Investigaciones en Ciencias de la Salud, Universidad Nacional de Asuncion, Asuncion, Paraguay; 4 Unidad de Epidemiología Molecular (UEM), Instituto de Patología Experimental, Universidad Nacional de Salta, Salta, Argentina; 5 Instituto de Medicina Tropical, Universidad Central de Venezuela, Los Chaguaramos, Caracas, Venezuela; Yale University, United States of America

## Abstract

**Background:**

Multilocus sequence typing (MLST) is a powerful and highly discriminatory method for analysing pathogen population structure and epidemiology. *Trypanosoma cruzi*, the protozoan agent of American trypanosomiasis (Chagas disease), has remarkable genetic and ecological diversity. A standardised MLST protocol that is suitable for assignment of *T. cruzi* isolates to genetic lineage and for higher resolution diversity studies has not been developed.

**Methodology/Principal Findings:**

We have sequenced and diplotyped nine single copy housekeeping genes and assessed their value as part of a systematic MLST scheme for *T. cruzi*. A minimum panel of four MLST targets (*Met-III*, *RB19*, *TcGPXII,* and *DHFR-TS*) was shown to provide unambiguous assignment of isolates to the six known *T. cruzi* lineages (Discrete Typing Units, DTUs TcI-TcVI). In addition, we recommend six MLST targets (*Met-II, Met-III, RB19, TcMPX, DHFR-TS,* and *TR*) for more in depth diversity studies on the basis that diploid sequence typing (DST) with this expanded panel distinguished 38 out of 39 reference isolates. Phylogenetic analysis implies a subdivision between North and South American TcIV isolates. Single Nucleotide Polymorphism (SNP) data revealed high levels of heterozygosity among DTUs TcI, TcIII, TcIV and, for three targets, putative corresponding homozygous and heterozygous loci within DTUs TcI and TcIII. Furthermore, individual gene trees gave incongruent topologies at inter- and intra-DTU levels, inconsistent with a model of strict clonality.

**Conclusions/Significance:**

We demonstrate the value of systematic MLST diplotyping for describing inter-DTU relationships and for higher resolution diversity studies of *T. cruzi*, including presence of recombination events. The high levels of heterozygosity will facilitate future population genetics analysis based on MLST haplotypes.

## Author Summary

The single-celled parasite *Trypanosoma cruzi* occurs in animals and insect vectors in the Americas. When transmitted to humans it causes a major public health problem, Chagas disease (American trypanosomiasis). *T. cruzi* is genetically diverse and currently split into six groups, known as TcI to TcVI. Multilocus sequence typing (MLST) is a method used for studying the population structure and diversity of pathogens. MLST involves sequencing the DNA of several different genes and comparing the sequences between isolates. MLST has not yet been developed and systematically applied to *T. cruzi*. He, we sequence nine *T. cruzi* genes, selecting a panel of four for lineage assignment and six for higher resolution studies of genetic diversity. Our results showed that one of the *T. cruzi* genetic groups is further subdivided into North and South American subpopulations. Furthermore, comparative analyses of the gene sequences gave new evidence of genetic exchange in *T. cruzi*. Application of MLST for assigning field isolates of *T. cruzi* to genetic groups and for detailed investigation of diversity provides a valuable approach to understanding the taxonomy, population structure, genetics, ecology and epidemiology of this important human pathogen.

## Introduction

*Trypanosoma cruzi* is the causative agent of Chagas disease and the most important parasitic infection in Latin America. Approximately 8 million people are thought to be infected [1]. Chagas disease is a zoonosis: *T. cruzi* infects many mammal species [2] and is transmitted to humans primarily by the infected faeces of haematophagous triatomine bugs coming into contact with mucosal membranes or broken skin. Transmission may also be by blood transfusion, congenitally or, rarely, by ingestion of food contaminated by infected triatomine faeces [3].

*T. cruzi* is monophyletic but genetically heterogeneous with at least six phylogenetic lineages (discrete typing units, DTUs) previously designated TcI, and TcIIa-e. A recent review meeting on *T. cruzi* intraspecific nomenclature reached an international consensus that these six DTUs should be renamed (former nomenclature in parenthesis): TcI (TcI), TcII (TcIIb), TcIII (TcIIc), TcIV (TcIIa), TcV (TcIId) and TcVI (TcIIe), to remove the presumptive sublineage designations within TcII [4]. Here we will adopt this new consensus nomenclature.

TcI and TcII are the most genetically distant groups. The evolutionary ancestry of TcIII and TcIV is presently a debated issue. Based on sequencing of individual nuclear genes Westernberger et al. [5] suggested an ancient hybridisation event occurred between TcI and TcII followed by a long period of clonal propagation leading to the extant DTUs TcIII and TcIV. Alternatively, de Freitas et al. [6] suggested that TcIII and TcIV have a separate evolutionary ancestry with mitochondrial sequences that are similar to each other but distinct from both TcI and TcII. Less controversially it is clear, using an array of molecular markers [6], [7], [8] that TcV and TcVI are hybrid lineages sharing haplotypes from both TcII and TcIII, with both DTUs retaining the mitochondrial genome of TcIII. Production of experimental hybrids [9] proved that *T. cruzi* has an extant capacity for genetic exchange (at least within TcI) but the mechanism of recombination is not fully understood, appearing to involve nuclear fusion followed by genome erosion leading to relatively stable aneuploid hybrids, a process distinct from classical meiosis [10].

There is currently no consensual discriminatory typing method applicable to *T. cruzi*. Previous methods for characterisation have included multilocus enzyme electrophoresis [11], [12], [13], [14], PCR amplification of single gene loci, [15] and various PCR based assays [16], [17]. Lewis et al. [18] recommended the use of a triple-assay comprising SSU rDNA, *HSP60* and *GPI* markers, allowing reliable and low cost typing to DTU level. Microsatellite typing (MLMT) provides a high resolution method for fine scale population genetics analysis [19]. What is still lacking is an unequivocally reproducible and standardised method that can simultaneously distinguish the known genetic lineages, describe inter-DTU relationships, and define high resolution intra-DTU diversity for population genetics studies.

Multilocus sequence typing (MLST) has been applied to a variety of bacterial [20], [21] and yeast species [22], [23] and typically involves sequencing internal fragments of six single copy housekeeping genes per strain [24]. The method is potentially highly discriminatory, when sequence polymorphisms within each housekeeping locus are categorised as distinct alleles. Sequence data for all loci are usually concatenated to produce a unique allelic profile (sequence type, ST). A major advantage of MLST analysis is that sound sequence data are unambiguous and suitable for population structure and epidemiological studies. Results are particularly relevant if easily accessible international databases are constructed such as MLST.net [25] which contains MLST typing schemes and data for a growing number of pathogens.

MLST was originally designed for haploid species, *T. cruzi* is diploid organism and as such heterozygosity renders MLST analysis more complicated. Heterozygosity from electropherograms can be inferred by a double peak (with two bases) at the same variable bi-allelic site [23]. One consequence of multiple bi-alleic sites is that of ambiguous allelic phase within loci and also ambiguous combinations of alleles across separate loci. However, it is possible for diploid sequence data, (without phase resolution) to be modified, concatenated across multiple loci [26] and applied in distance based phylogenetic methods for lineage assignment (see Materials and Methods). From these data one can also infer evolutionary relatedness, and detect gene mosaics, within or between homozygous gene loci [27]. A MLST approach specific to *T. cruzi* was first utilised by Machado and Ayala using two nuclear loci [8] in their study of the genetic recombination in natural *T. cruzi* populations and this is now being expanded by others to additional targets [28]. However the use of multicopy target genes is not recommended due to the possibility of non-identical paralogous copies confusing phylogenetic signals. The repetitive nature of the *T. cruzi* genome has previously hampered the search for suitable single copy targets; at least 50% of the *T. cruzi* genome consists of tandemly repeated genes [29]. However, the recent publication of a draft chromosomal level assembly by Weatherly et al. [30] has enabled both the determination of copy number and chromosomal distribution of markers to be established with reasonable confidence. A standardised panel of suitable MLST gene loci has yet to be developed for *T. cruzi*. Here we address this omission by sequencing and assessing 9 nuclear targets and evaluating them with a cohort of reference strains representing the known lineages. We demonstrate the potential of this formalised MLST for describing lineage assignment, describing inter-DTU relationships and for high resolution population genetic analysis of *T. cruzi*.

## Materials and Methods

### Isolates

A panel of 39 individual isolates (Table 1) was assembled, consisting of cloned reference strains encompassing all of the known DTUs (Table 1) spanning wide geographical and varied ecological origins. Isolates were previously characterised to DTU level by amplified fragment length polymorphisms (RFLP) in the D7 divergent domain of the 24Sα rRNA, and restriction fragment length polymorphism (RFLP) in the heat shock protein 60 (*HSP60*) and *GPI* genes [18]. Parasites were cultivated in supplemented RPMI liquid medium at 28°C, as described previously [31]. Genomic DNA was prepared from logarithmic phase cultures using Dneasy kits (Qiagen, UK).

**Table 1:** Cohort of reference clonal isolates representing the six known *T. cruzi* lineages (DTUs).

Strain	DTU	Origin	Host
C8 cl1	TcI	La Paz, Bolivia	*Triatoma infestans*
X10/1	TcI	Belém, Brazil	*Homo sapiens*
JR cl4	TcI	Anzoategui, Venezuela	*Homo sapiens*
PI (CJ007)	TcI	Carajas, Brazil	*Didelphis marsupialis*
PII (CJ005)	TcI	Carajas, Brazil	Unidentified triatomine
B187 cl10	TcI	Pará State, Brazil	*Didelphis marsupialis*
SAXP18 cl1	TcI	Majes, Peru	*Didelphis marsupialis*
92101601P cl1	TcI	Georgia, U.S.A.	*Didelphis marsupialis*
Esm cl3	TcII	Sáo Felipe, Brazil	*Homo sapiens*
Pot7a cl1	TcII	San Martin, Paraguay	*Triatoma infestans*
Pot7b cl2	TcII	San Martin, Paraguay	*Triatoma infestans*
Tu18 cl2	TcII	Tupiza, Bolivia	*Triatoma infestans*
Chaco23 col4	TcII	Chaco, Paraguay	*Triatoma infestans*
M5631 cl5	TcIII	Marajo, Brazil	*Dasypus novemcinctus*
M6421 cl6	TcIII	Belém, Brazil	*Homo sapiens*
ARMA 18 cl3	TcIII	Camp Lorro, Paraguay	*Dasypus novemcinctus*
ARMA 13 cl1	TcIII	Campo Lorro, Paraguay	*Dasypus novemcinctus*
JA2 cl2	TcIII	Amazonas, Brazil	Unknown
CM25 cl2	TcIII	Carimaga, Colombia	*Dasyprocta fuliginosa*
85/847 cl2	TcIII	Alto Beni, Bolivia	*Dasypus novemcinctus*
SABP19 cl5	TcIII	Peru	*Triatoma infestans*
StC10R cl1	TcIV	Georgia, U.S.A.	*Procyon lotor*
92122102R	TcIV	Georgia, U.S.A.	*Procyon lotor*
10R26	TcIV	Santa Cruz, Bolivia	*Aotus* Sp.
CanIII cl1	TcIV	Belém, Brazil	*Homo sapiens*
Saimiri3 cl1	TcIV	Venezuela	*Saimiri sciureus*
PARA4 cl3	TcV	Paraguari, Paraguay	*Triatoma infestans*
PARA6 cl4	TcV	Paraguari, Paraguay	*Triatoma infestans*
Sc43 cl1	TcV	Santa Cruz, Bolivia	*Triatoma infestans*
92–80 cl2	TcV	Santa Cruz, Bolivia	*Homo sapiens*
Chaco2 cl3	TcV	Chaco, Paraguay	*Triatoma infestans*
Vinch101 cl1	TcV	Limari, Chile	*Triatoma infestans*
PAH179 cl5	TcV	Chaco, Argentina	*Homo sapiens*
CL Brener	TcVI	Rio Grande do Sul, Brazil	*Triatoma infestans*
Tula cl2	TcVI	Tulahuen, Chile	*Homo sapiens*
P251 cl7	TcVI	Cochabamba, Bolivia	*Homo sapiens*
EPV20-1 cl1	TcVI	Chaco, Argentina	*Triatoma infestans*
LHVA cl4	TcVI	Chaco, Argentina	*Triatoma infestans*
VFRA1 cl1	TcVI	Francia, Chile	*Triatoma infestans*

### Choice of loci

Initially, 11 genes were investigated. This number was subsequently reduced to 9 single copy targets after screening for reliability of PCR amplification. The excluded genes were trypanothione-dependent glyoxalase I (gene ID, Tc00.1047053510659.240)and cyclophilin (gene ID, Tc00.1047053510947.50). Targets were verified by PCR amplification and sequences submitted for BLAST (blastn) analyses hosted at NCBI. Copy number of targets was verified by submission of gene IDs to TriTyrpDB 2.2 (http://TriTrypDB.org). The 9 single copy gene fragments amplified for MLST analysis were ascorbate-dependent haemoperoxidase (*TcAPX*), dihydrofolate reductase-thymidylate synthase (*DHFR-TS*), glutathione-dependent peroxidase II (*TcGPXII*), mitochondrial peroxidase (*TcMPX*), trypanothione reductase (*TR*), RNA-binding protein-19 (*RB19*), metacyclin-II (*Met-II*), metacyclin-III (*Met-III*) and *LYT1*. The ratio of nonsynonymous to synonymous amino acid changes (dN/dS) was calculated according to the Nei-Gojobori method [32] using SNAP software available at http://www.hiv.lanl.gov, [33] to infer relative selection pressures. Genes possessing a dN/dS ratio <1 meet the criteria for stabilising selection for the conservation of metabolic function. Taylor & Fisher [34] recommended the incorporation of some loci with a dN/dS ratio of >1 in order to obtain sufficient sequence diversity.

### PCR amplification

Primers and annealing temperatures for PCR amplification are given in Table 2. For *DHFR-TS* and *TR*, cyclic amplifications were performed with an initial denaturation step for three minutes at 94°C, followed by 30 amplification cycles (94°C for 30 seconds, 58°C for 1 minute, 72°C for 2 minutes). Annealing temperatures were 55°C for *TcAPX,* 50°C for *TcMPX* and 62°C *TcGPXII*. Reaction conditions for *Met-II*, and *RB19* were as follows: 3 minutes at 94°C, followed by 30 amplification cycles (94°C for 30 seconds, 53°C for 30 seconds, 72°C for 45 seconds; annealing temperatures were 51°C for *Met-III* and 56°C for *LYT1*). All reactions had a final ten minute elongation step at 72°C. Each 20 µl total reaction volume contained: 125 ng genomic DNA, 1 µl of each primer (20 pmol/µl), 2 µl dNTPs (2 mM), 0.8 µl (50 mM) MgCl_2_ and 5U *Taq* (BIO-21086, Bioline, UK).

**Table 2:** Details of gene targets.

Gene	Gene ID	Chromosome Number	Primer Sequence (5′→3′)	Annealing Temp. (C)	Amplicon Size (bp)	Sequence Start 5′	Sequence Start 3′	Fragment Length (bp)
*LYT1*	Tc00.1047053508045.40	22	CAACTTGCCCTTTTGCTCTC (20)	56	804	TTTGTGAC	GTGGTGGA	691
			GTTGTTGTTGTTGCCCTGTG (20)					
			ACAAGGGTGTTTCCGTGAAG (20)					
*Met-II*	Tc00.1047053510889.28	6	TCATCTGCACCGATGAGTTC (20)	53	702	CATTTTCA	TTTTGCCA	390
			CTCCATAGCGTTGACGAACA (20)					
			TGTGTCCACAATTCGTCGTT (20)					
*Met-III*	Tc00.1047053510943.44	36	GTGGCTCCAAAGGCATAAGA (20)	51	824	TTCATCCG	TTTCTTTG	619
			CCCCCTTCTTTGCCAATTAT (20)					
			CACATGAGGTCGTGTTGTCC (20)					
*RB19*	Tc00.1047053507515.60	29	GCCTACACCGAGGAGTACCA (20)	53	408	GTCGTGCG	CCCAGACT	350
			TTCTCCAATCCCCAGACTTG (20)					
*TcAPX*	Tc00.1047053506193.60	36	GGGAAGCTTTCACTTTGCCTATTTTGACTC (30)	55	1500	GCGAGTCG	GGCGCCGG	799
			GGGGGATCCATGGCTTTTTGTTTTGGTTCA (30)					
*TcMPX*	Tc00.1047053509499.14	22	ATGTTTCGTCGTATGGCC (18)	50	678	TACATGGA	CGCACCGT	505
			TGCGTTTTTCTCAAAATATTC (21)					
*DHFR-TS*	Tc00.1047053509153.90	27	CGCTGTTTAAGATCCGNATGCC (22)	58	1473	GCGGGAGA	CACGCTCT	715
			CGCATAGTCAATGACCTCCATGTC (24)					
			ACCCTGTCCGTCATAGTTG (19) INT					
*TcGPXII*	Tc00.1047053511019.99	35	GGGCAGCAGCACGCTGTTCG (20)	62	487	TTGCGGCC	ATCCGGCC	437
			TCATGCACCCCGTTGCGGCCC (21)					
*TR*	Tc00.1047053503555.30	37	ACTGGAGGCTGCTTGGAACGC (21)	58	1290	TGTCAATG	TACGAAGG	602
			GGATGCACACCRATRGTGTTGT (22)					
			CGAATGARGCATTYTACCTG (20) INT					
			TACTCGTCCACCTGCACACCAC (22) INT					

PCR products were purified with QIAQuick PCR purification kits (Qiagen) or SureClean (Bioline, UK). Bi-directional sequencing was performed with Big Dye Terminator Cycle Sequencing V3.1 (Applied Biosystems) in ABI PRISM 377 DNA Sequencers (Applied Biosystems) according to the manufacturer's protocol. Gene fragments were sequenced in both directions (5′ and 3′) with the PCR primers described in Table 2. Additionally, in the case of *LYT1*, *Met-II*, *Met-III*, *DHFR-TS* and *TR* internal primers were used to obtain full sequence coverage. Sequence data were assembled manually in BioEdit v7.0.9.0 sequence alignment editor software (Ibis Biosciences, USA) and ambiguous peripheral regions of aligned sequences discarded to produce unambiguous partial gene sequences for each isolate. Chromatograms were examined visually in both directions and in most instances the results easily interpreted as heterozygous when two peaks in a chromatogram overlap. Re-sequencing was undertaken if results were ambiguous.

### Strain differentiation by diploid sequence typing

The number of sequence types (STs) for each gene fragment was identified from SNP data across the panel of isolates and the discriminatory power (DP, genotypes recovered per unique isolate tested) for each gene determined. STs were conjoined across gene fragments in order to identify a diploid sequence type (DST) and to assess overall discriminatory power (DP). DSTs were applied to eBURST software to infer evolutionary relationships and founders. The eBURST algorithm (http://eburst.mlst.net) identifies related sequences and predicts a founding genotype (based on the most overrepresented genotype) with variants identified depending on the number of different loci.

Phylogenetic analysis was performed on modified sequences using the methodology described by Tavanti et al. [26] which renders diplotypic data suitable for use in MEGA analysis [35] to produce distance based dendrograms. Briefly, variable loci between isolates can be considered either homozygous or heterozygous, assuming that *T. cruzi* is minimally diploid. For example, a homozygous variable locus scored as A (adenine) was modified by duplication to AA, and a heterozygous locus, for example Y (C or T, in accordance with IUPAC nomenclature), scored as CT, effectively creating a difference matrix across the panel. Phylogenetically the data were examined in two different ways. Firstly sequences were examined at the level of individual genes by generating neighbor-joining trees (MEGA v4.0.2) in order to assess the “usefulness” of gene fragments in the context of lineage assignment, intralineage resolution and associated bootstrap values. Secondly, sequence data were concatenated across multiple gene fragments to produce MLST neighbor-joining trees.

Analysis of recombination at the level of individual genes was applied to isolates with unambiguous phase applied through the software package RDP3 [36] incorporating the following methods: RDP [37], Bootscanning [38], GENECONV [39], Maximum Chi Square method [40], [41], the Chimaera method [40], the Sister Scanning Method [42], the 3SEQ method [43], the Reticulate compatibility matrix method [44] and the TOPAL DSS method [45], [46],

## Results

### Chromosomal localisation of markers

Gene IDs were confirmed by BLAST searches and submitted to the TriTypDB 1.3 (http://TriTrypDB.org) to identify chromosomal location. Locations for each of the the genes under study are shown in Table 2. Two pairs of genes were physically linked: genes *LYT1* (367579–369237) and *TcMPX* (P:120685–121365) on chromosome 22, and *MET-III* (P:945156–945731) and *TcAPX* (P:1142201–1143187) on chromosome 36. The remaining genes were on independent chromosomes. The ratios of nonsynonymous to synonymous amino acid substitutions, resulting from sequence polymorphisms, were 1.0 or less for 7 genes, two genes possessed dN/dS ratios of above 1 (Table 3).

**Table 3:** Properties of nine *T. cruzi* MLST targets.

Gene Fragment	No. Of Polymorphic Sites	No. Of Genotypes	No. Of Genotypes/Polymorphism(Typing Efficiency)	Ratio Of Nonsynonymous To Synonymous Changes
*LYT1*	47	22	0.47	0.833
*Met-II*	51	24	0.47	0.880
*Met-III*	50	24	0.48	0.440
*RB19*	27	24	0.89	0.129
*TcAPX*	27	14	0.52	2.04
*TcMPX*	15	16	1.06	0.061
*DHFR-TS*	32	19	0.59	0.088
*TcGPXII*	27	18	0.66	0.502
*TR*	28	21	0.75	1.964

### Nucleotide polymorphisms and amino acid changes

The 304 nucleotide polymorphisms among the nine sequenced fragments resulted in 121 nonsynonymous changes in amino acids encoded by sequence-variable triplets. Up to three different amino acids were present in different isolates at the same locus across the panel. A deletion of 3 nucleotides (AAA  =  Lysine at position 179–181) was present in C8, X10/1 and SAXP18 (TcI) within *LYT1*. Within *TcMPX*, a single non-synonymous nucleotide polymorphism resulted in a conservative amino acid change between an aspartic acid residue and a glutamic acid residue was evident (position 21). All TcV representatives possessed glutamic acid but all other panel isolates (including TcVI) possessed aspartic acid residues at the corresponding position.

### Discriminatory power of MLST targets by diploid sequence typing

Diploid sequence typing using 9 genes was able to discriminate 38 of 39 isolates (DP 0.97, Table 4). Importantly, using only 6 genes (*Met-II*, *Met-III*, *RB19*, *TcMPX*, *DHFR-TS* and *TR*) the same number of DSTs could be identified. Only two isolates, Sc43 and Vinch101, shared a DST (29). Both are positioned within the DTU TcV which is known to possess a particularly homogenous population structure by microsatellite analysis [10]. All other isolates, notably even those those within the relatively homogenous clade TcVI, produced a unique isolate specific DST. Discriminatory power was determined for each of the 9 gene fragments (Table 4), in decreasing power, as follows: *Met-II*, *Met-III* and *RB19* (0.59), *LYT1* (0.56), *TR* (0.54), *DHFR-TS* and *TcGPXII* (0.49), *TcMPX* (0.41), and *TcAPX* (0.38). No single gene was able to distinguish all 39 reference strains. *Met-II, Met-III* and *RB19,* taken individually, were the most discriminatory genes, all three identifying 24 separate genotypes from the panel of 39. *TcAPX* was the least resolutive marker distinguishing 15 of 39 isolates (DP 0.38). Table 4 shows in detail the number of STs (sequence types) that each individual gene fragment resolved, and also the derived DSTs obtained from the concatenation of all 9 STs for each isolate. Table 4 also indicates the reduced panel of 6 genes required to obtain the same 38 DSTs. eBURST analysis of the genotypes and DSTs for 39 *T. cruzi* isolates from the panel revealed one cluster of 7 DSTs within TcV as the only related set to emerge from this analysis. Vinch101 (Limari, Chile) and Sc43 (Santa Cruz, Bolivia), both DST 29 (Table 4) are the predicted genetic founders of isolates of all other TcV isolates. The remaining isolates across other DTUs appeared as unrelated singletons (isolates that do not belong to any cluster). Overall the results reveal that diploid sequence typing using just 6 genes is highly discriminatory.

**Table 4:** Sequence types (STs) and diploid sequence types (DSTs) for nine gene fragments.

Strain	DTU	DST	*LYT1* (ST)	*Met-II* *	*Met-III* *	*RB19* *	*TcAPX*	*TcMPX* *	*DHFR-TS* *	*TcGPXII*	*TR* *
C8 cl1	TcI	1	1	1	1	1	1	1	1	1	1
X10/1	TcI	2	2	2	1	2	2	1	1	2	1
JR cl4	TcI	3	3	3	2	3	3	1	1	3	1
PI (CJ007)	TcI	4	4	1	1	4	2	1	1	1	2
PII (CJ005)	TcI	5	5	4	3	1	2	1	1	1	3
B187 cl10	TcI	6	6	4	1	5	2	1	1	4	4
SAXP18 cl1	TcI	7	1	1	1	6	2	1	1	1	5
92101601P cl1	TcI	8	7	1	4	7	2	1	2	3	6
Esm cl3	TcII	9	8	5	5	8	4	2	3	5	7
Pot7a cl1	TcII	10	8	5	6	8	4	3	4	5	7
Pot7b cl2	TcII	11	8	5	5	8	4	3	4	5	8
Tu18 cl2	TcII	12	9	6	7	9	5	2	5	5	8
Chaco23 col4	TcII	13	10	7	8	9	4	2	6	6	8
M5631 cl5	TcIII	14	11	8	9	10	6	4	7	7	9
M6421 cl6	TcIII	15	11	9	10	11	7	4	7	8	10
ARMA18 cl3	TcIII	16	12	10	10	12	6	5	7	8	11
ARMA13 cl1	TcIII	17	12	9	11	13	6	5	7	9	11
JA2 cl2	TcIII	18	13	11	12	14	6	4	8	10	10
CM25 cl2	TcIII	19	14	12	13	15	7	4	7	11	12
85/847 cl2	TcIII	20	15	11	14	16	6	4	9	12	13
SABP19 cl5	TcIII	21	16	13	15	17	6	6	7	7	11
StC10R cl1	TcIV	22	17	14	16	18	8	7	10	13	14
92122102R	TcIV	23	17	15	17	18	8	8	11	14	15
10R26	TcIV	24	18	16	18	19	9	9	12	15	16
Can III cl1	TcIV	25	19	17	19	20	10	10	13	16	17
Saimiri3 cl1	TcIV	26	20	18	20	20	11	11	14	17	18
PARA4 cl3	TcV	27	21	19	21	21	12	11	15	18	19
PARA6 cl4	TcV	28	21	20	22	21	12	11	15	18	19
Sc43 cl1	TcV	29	21	19	22	21	13	11	16	18	19
92–80 cl2	TcV	30	21	19	22	21	13	11	15	18	19
Chaco2 cl3	TcV	31	21	19	22	21	13	11	16	18	20
Vinch101 cl1	TcV	29	21	19	22	21	13	11	16	18	19
PAH179 cl5	TcV	32	21	19	22	21	14	12	16	18	19
CL Brener	TcVI	33	22	21	23	22	13	13	17	18	19
Tula cl2	TcVI	34	22	21	24	23	13	14	18	18	19
P251 cl7	TcVI	35	22	21	24	22	13	8	18	18	19
EPV20–1 cl1	TcVI	36	22	22	23	22	13	15	18	18	19
LHVA cl4	TcVI	37	22	23	23	22	14	8	18	18	19
VFRA1 cl1	TcVI	38	22	24	23	24	15	16	19	18	21
Discriminatory Power		0.97	0.56	0.62	0.62	0.62	0.38	0.41	0.49	0.49	0.54

*Indicate six genes required to identify the same number of DSTs as the full panel of 9 genes.

### Intra DTU diversity

Table 5 describes the levels of diversity seen in each gene fragment represented as the number of variable sites per DTU (VS), the number of genotypes differentiated per DTU (GT) and the discriminatory power for each gene fragment for each DTU (DP). The number of genotypes identified at the intra DTU level, varied widely and is correlated to the SNP diversity within each gene fragment. The most and least discriminatory genes for each of the six DTUs are also shown in detail in Table 5. *LYT1* and *RB19* genes revealed the most diversity for TcI discriminating 7 of 8 isolates (DP 0.88). Two genes *DHFRS-TS* and *Met-III* gave the highest resolution for TcII (DP 0.80), generally considered to be a relatively homogenous clade, and revealed surprising discriminatory ability, distinguishing 4 of 5 isolates. A single gene (*RB19*) was able to distinguish all 8 reference isolates within TcIII (DP 1.0). Six of 9 genetic loci were able to discriminate individually the full panel of 5 TcIV reference strains (DP 1.0). DTUs TcV and TcVI have previously been shown to have homogenous population structures with low intralineage diversity. Nevertheless *TcAPX* resolved three of 5 isolates within TcV (DP 0.43) and *TcMPX* 5 of 6 (DP 0.83) isolates within DTU VI.

**Table 5:** Intra-lineage diversity.

SNPs per DTU	No. Isolates	*LYT1*			*Met-II*			*Met-III*			*RB19*			*TcAPX*			*TcMPX*			*DHFR-TS*			*TcGPXII*			*TR*		
		VS	GT	DP	VS	GT	DP	VS	GT	DP	VS	GT	DP	VS	GT	DP	VS	GT	DP	VS	GT	DP	VS	GT	DP	VS	GT	DP
TcI	8	8	7	0.88	10	4	0.50	6	4	0.50	7	7	0.88	2	3	0.38	0	1	0.13	1	2	0.25	4	4	0.50	5	6	0.75
TcII	5	6	3	0.60	3	3	0.60	5	4	0.80	1	2	0.40	1	2	0.40	1	2	0.40	10	4	0.80	2*	2	0.40	2	2	0.40
TcIII	8	7	6	0.75	7	6	0.75	12	7	0.88	7	8	1.0	1	2	0.25	1	3	0.38	4	3	0.38	5	6	0.75	5	5	0.63
TcIV	5	13	4	0.80	10	5	1.0	10	5	1.0	3	3	0.6	5	4	0.80	6	5	1.0	5	5	1.0	8	5	1.0	7	5	1.0
TcV	7	0	1	0.14	18	2	0.29	1	2	0.29	0	1	0.14	5	3	0.43	5*	2	0.28	1	2	0.29	0	1	0.14	1	2	0.29
TcVI	6	0	1	0.17	14	4	0.67	20	2	0.33	8	3	0.50	4	2	0.33	5	5	0.83	2	3	0.50	0	1	0.16	1	2	0.33

Abbreviations in each of the gene fragment columns are as follows: VS = No variable sites per DTU, GT = Number of genotypes per DTU, DP = Discriminatory power. Numbers in bold indicate highest DP values per DTU. *All SNP variation observed within the DTU originated from a single isolate.

### Phylogenies and incongruence

**Figure 1:**
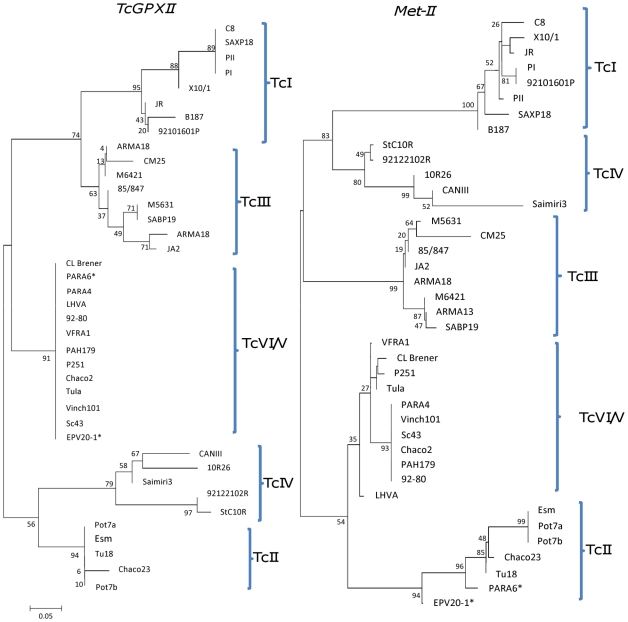
Phylogenetic DTU incongruence between two individual gene trees (TcI, TcIII and TcIV). *loss of heterozygosity in isolates at the *Met-II* locus (EPV20-1, TcVI: Para 6, TcV).

**Figure 2:**
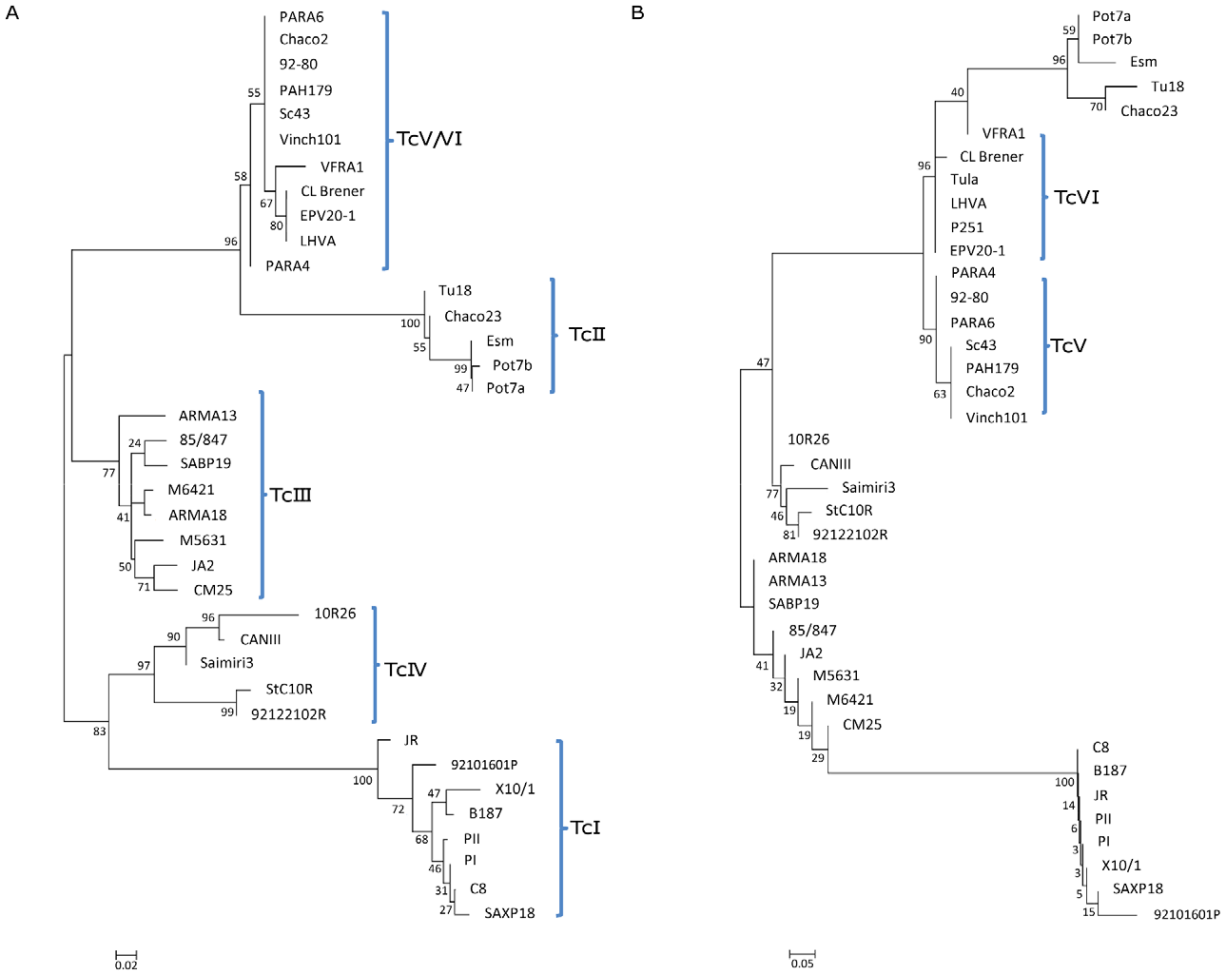
Concatenation and lineage assignment. A combination of 4 genes allows for DTU assignment. 2A, Unrooted neighbor-joining diplotypic tree showing *p*-distance for 3 concatenated gene fragments (*Met-III*, *RB19,* and *TcGPXII*) which discriminate all lineages except TcV and VI. 2B, *DHFR-TS* differentiates between TcV and TcVI.

In addition to the derivation of DSTs, neighbor-joining trees for individual and concatenated gene fragments were constructed, representatives of which are shown in Figures 1 and 2. Concatenation using all of the original 9 gene fragments generated phylogenies with the expected DTU assignments for all 39 isolates with much higher bootstrap values than for individual genes (supporting information, Figure S1); there were no unexpected outliers. However, bootstrap support distinguishing TcV and VI was low (21%), but to distinguish between these DTUs concatenation of just two genes (*DHFR-TS* and *LYT1* sequences) generated robust lineage assignment, and also two separate clusters within TcV and TcVI (supporting information, Figure S1). Generally, individual trees assigned each of the reference DTUs to the predicted clade (TcI-TcIV). As expected, bootstrap support for individual gene loci was variable depending on locus. For example *TcAPX*, generated dendrograms with high bootstraps for lineage assignment (with the exception of TcV and TcIV) but low intralineage diversity. Other genes (for example *Met-II* and *Met-III*) generated trees with high bootstrap values for both lineage assignment and comparatively high bootstraps for intralineage diversity. Conversely, Tc*MPX* produced the least phylogenetically informative dendrograms with low associated bootstrap values. Three genes (*LYT1*, *DHFRS-TS*, *RB19*) individually separated the difficult to distinguish homogenous clades TcV and TcVI with relatively high bootstraps. Loss of heterozygosity (LOH, the loss of one allele at a specific locus) was observed in DTUs TcV and TcVI, in some isolates, in 4 gene fragments (see below) and confused the phylogenetic assignment of those individual isolates affected. In these instances the remaining allelic profiles clustered with either the respective TcII or TcIII clades. One surprising output was the level of incongruent DTU topologies between individual genetic loci (Figure 1). For example *TcAPX*, *TcMPX*, *Met-II*, and *Met-III* generated TcI clades that were most closely associated to TcIV. In contrast, *DHFR-TS, TR,* and *LYT1* dendrograms inferred a closer association of TcI to TcIII. *TcGPXII* inferred that TcI was more similar to TcIII. Similarly, at the intra DTU level there was also incongruence between dendrograms. For example, *TcGPXII* and *Met-II* each separated TcIII into two clusters but with incongruent isolates (for example M5631 and CM25, Figure 1).

### Choice of loci for lineage assignment

The minimum number of genes required for phylogenetic assignment to DTU level was investigated according to the following criteria. Firstly, the minimum number of genes required to produce phylogenetically robust bootstrap support. Secondly, to choose those genes that produced the most consistent unambiguous sequences. Thirdly, to choose genes requiring the minimum number of internal primers. Using these criteria a minimum combination of 4 genes (*Met-III*, *RB19*, *TcGPXII* and *DHFR-TS*) enabled all reference isolates to be assigned to DTU level. Specifically, concatenated sequences of *Met-III*, *RB19* and *TcGPXII* assigned isolates to DTUs TcI-TcIV (Figure 2A). Separately, DHFR-TS (the only gene requiring internal primers) distinguished the DTUs TcV and TcVI (Figure 2B, insert). Internal primers for *Met-III*, *RB19* and *TcGPXII* were not required after initial optimisation (Table 2).

### Intralineage phylogenies

Generally, bootstrap support within DTUs using concatenated sequences was lower than between DTUs (Figure 1) which is expected in data originating from gene fragments with non-contiguous topologies at the intralineage level. In most instances intralineage topology of individual gene dendrograms was generally low and/or not contiguous across loci (Figure 1). However, certain observations deserve closer scrutiny. Importantly, two distinct clusters within TcIV separated isolates from North America and South America. This genetic partitioning was detected in 8 out of 9 gene individual targets in the present study, indicating a robust cladistic subdivision within TcIV. Interestingly, two TcI isolates SAXP18 (Peru, *Didelphis marsupialis*) and C8 (Bolivia, *Triatoma infestans*) were isolated from silvatic and domestic sources respectively, and are genetically similar suggesting present or past overlapping transmission cycles. Relatively high bootstrap support for isolates of the TcII clade did not reveal evidence of geographical clustering and genetically similar isolates originated from distant localities. Within TcIII at least two clusters were seen (in concatenated data); silvatic isolates (from *Dasypus novemcinctus*) in the Paraguayan Chaco clustered with a domestic strain (SABP19) isolated from a domestic *T. infestans* in Peru. The 8 TcIII isolates included in our panel showed considerable diversity. However, numbers are insufficient to examine population substructuring.

### Intralineage recombination

**Figure 3:**
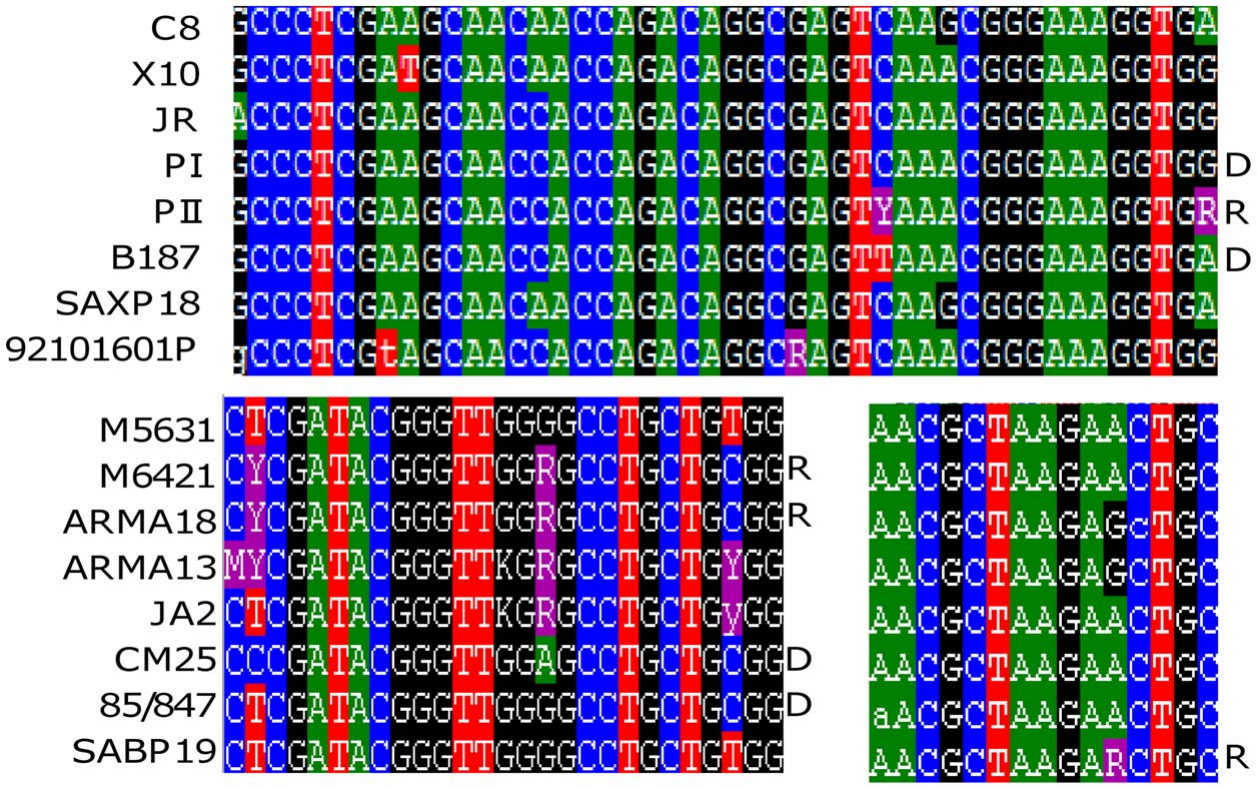
Raw SNP data showing putative donor and recipient isolates. Sequences containing heterozygous SNP's (R) and putative homozygous donors (D) within a single DTU. Genetic loci are *LYT1* (top), *TcGPXII* (bottom left) and *TcMPX* (bottom right).

Analysis for the presence of mosaic alleles, at the level of individual genetic loci was performed using RDP [37], applied to a total of 240 representative sequences of known allelic phase (single SNP heterozygous and homozygous sequence profiles) encompassing DTUs TcI, TcII, TcIII and TcIV across the 9 genetic loci. We found no evidence of allelic mosaics within individual genes. However, diplotypic SNP data revealed for three genes (*LYT1*, *TcGPXII* and *TcMPX)* that putative donor homozygous SNP profiles and the corresponding heterozygous profiles were present as shown in Figure 3. Specifically, within *LYT1* the heterozygous isolate (PII) and putative donors (B187 and PI) were identified within TcI. For *TcGPXII*, 2 heterozygous isolates (ARMA18 and M6421) possess SNP profiles of the putative donor isolates CM25 and 85/847 (TcIII). Lastly, for *TcMPX*, SABP19 was heterozygous at a single locus with potential donors in the same DTU (TcIII). In each instance individual isolates containing heterozygous and donor SNPs were present only in a single gene and were not contiguous across loci.

### SNP data and loss of heterozygosity

Our panel of isolates included 13 representatives of DTUs V and VI, which are known to be genetic hybrids of TcII and TcIII [8] Across all 9 loci the expected allelic heterozygous profile was observed in most individual isolates. However, at four gene loci complete LOH was detected for individual isolates within the hybrid lineages. The observations are as follows: within *Met-III*, two TcVI isolates (Tula cl2 and P251 cl7) possessed a TcII-like allele but not a TcIII-like allele as did an individual TcV isolate for *Met-II* (PARA6 cl4). In contrast a single TcVI isolate at the locus *RB19* (VFRA cl1) possessed only the TcIII-like allele. At the *TcMPX* locus, all TcV isolates (with the exception of PAH179) appeared to have lost the TcIII-like allele and 2 representatives of TcVI (P251 and LHVA) lost the TcII-like allele; the remaining 4 TcVI isolates retained heterozygosity. LOH among individual isolates in genetic loci could be clearly visualised when dendrograms were constructed, for example, hybrid isolates PARA6 (TcV) and EPV20-1(TcVI) for *Met-II* clustered within TcII (Figure 1). There was no continuity of allelic loss across different genes for individual isolates. For example, the two aforementioned isolates exhibiting homozygosity in *Met-III* were heterozygous in *TcAPX,* which are linked on chromosome 36. It is therefore evident that heterozygosity has been maintained elsewhere on the same chromosome. Taken together the results indicate a return to homozygosity in 6.24% of TcV and TcIV (combined) isolates when considering the four affected gene fragments.

## Discussion

### Discriminatory power and diploid sequence typing

Diploid sequence typing of 39 reference isolates indicates that together the nine genes under study were highly discriminatory, 38 of 39 reference isolates generated a unique DST. It was apparent that the number of STs identified for each gene fragment varied considerably (Table 5) despite the fact that all but two of the genes (*TcAPX* and *TR*) were under stabilising selection. However, by using just 6 of the original panel of nine genes the same 38 DSTs could be identified. The reduced panel consisted of those genes with the highest overall DPs (*Met-II*, *Met-III* and *Rb19*) with additional genes (*TcMPX*, *DHFR-TS* and *TR*) resolving further STs for the genetically homogenous DTUs TcV and TcVI. The reduction in the number of genes would represent a considerable saving in sequencing effort if applied to new samples. Furthermore, the reduced panel consists primarily of genes that are easily amplified and sequenced. The derived STs and DSTs applied to eBURST software, designed specifically for MLST data to infer evolutionary relationships and genetic founders, identified one cluster encompassing all of the TcV reference isolates. The inferred founder of this group (DST29) was found in 2 isolates (Vinch101 and Sc43). Both of these isolates were originally isolated from domestic *T. infestans* from Chile and Bolivia respectively. The ability to differentiate isolates within TcV is an important development, as this DTU is found throughout the Southern Cone countries in domestic cycles. Currently other methods to demonstrate diversity within TcV have been lacking. The high number of singletons observed in remaining DTUs was surprising, although this is probably a consequence of reference isolates spanning diverse spatial and ecological origins and diversity found within our panel. It is likely that high intensity localised sampling would reveal more easily genetic relationships between isolates. High numbers of singletons are often observed in other MLST typing schemes (for example *Candida* species) when applied to small datasets. Furthermore distance based dendrograms have been shown to correlate well with clonal clusters generated by eBURST when applied to large numbers [27]. It is also worth noting that high rates of singletons are typical of populations with a high rate of recombination relative to mutation [27]. Although eBURST is an established software for diplotyping and haplotyping pathogens [47], [48] until analysis has been applied to a larger number of sympatric *T. cruzi* isolates it is difficult to evaluate the usefulness of the analysis as a tool for predicting founders and clusters in DTUs other than TcV, and other approaches should be considered in parallel.

### Phylogenies and incongruence

A separate approach for the analysis of diplotypic MLST data is to generate phylogenetic trees utilising distance based methods and modified sequences, adapted for diplotyping. At the level of individual gene fragments, isolates were generally assigned to the predicted clades, although bootstap support varied widely and no single gene was able to assign all isolates robustly. A noticeable observation was that of incongruent topologies in individual gene trees and in particular between DTUs TcI, TcIII, and TcIV. At the intra DTU level there was incongruence between dendrograms (Figure 1). The bootstraps supporting incongruence varied widely between genes and DTUs and similar patterns of incongruence have been previously observed in nuclear genes [5], [49]. Specifically, our data revealed that DTUs TcIII and TcIV possessed split affinities to DTUs TcI and TcII. For 4 genes, DTUs TcIII and TcIV showed greater affinity to TcI. These split affinities of TcIII and TcIV to TcI and TcII are in broad agreement with the spectrum of polymorphism observed by Westenberger et al. [5]. Such incongruence is not immediately compatible with a model of strict clonality; genealogical relationships where sequences vary in topological position is a classical marker in populations that have undergone recombination.

### Choice of loci for lineage assignment

Sequence data concatenated across all 9 genes produced robust phylogenetic assignment for all DTUs except for TcV and TcVI, which could be resolved using two concatenated genes (*DHFR-TS* and *LYT1* sequences). Importantly, this number could subsequently be reduced to a combination of just 4 genes (*Met-III*, *RB19*, *TcGPXII* and *DHFR-TS*) while maintaining high bootstrap support. The first three aforementioned gene sequences were concatenated to differentiate DTUs TcI-TcIV, with *DHFR-TS* dendrograms separately able to distinguish DTUs TcV and VI. Notably concatenated data implies a cladistic subdivision within TcIV, separating North American from South American isolates. Bootstrap support is a requirement for any MLST scheme, but of equal importance are reliable PCR amplifications which generate unambiguous sequences in both forward and reverse directions. All four targets meet this criterion. *Met-III*, *RB19* and *TcGPXII* do not require internal primers, although some were used initially in the optimisation process, and generate small amplicons (824 bp, 408 bp and 487 bp respectively). *DHFR-TS* alone requires the use of internal sequencing primers. *LYT1* was considered to be an alternative candidate, also able to distinguish TcV and TcVI, but it was technically more difficult to obtain sequences in the forward direction despite multiple attempts at optimisation.

### Intralineage recombination

SNP data revealed that for three genes (*LYT1*, *TcGPXII* and *TcMPX)* putative donor homozygous SNP profiles and the corresponding heterozygous profiles, were present within TcI (a single heterozygous isolate) and TcIII (1 heterozygous isolate) one tentative explanation of the presence of heterozygous SNPs is that of a recombination signature within these DTUs. In the present paper examination 240 sequences (of known allelic phase) using an array of recombination detection algorithms did not detect evidence of allelic mosaics. This result is not unexpected as diplotyping is not as sensitive as haplotyping for detecting mosaic's or investigating the sexual reproduction in evolutionary history [50]. Future recombination analysis will include more refined phase resolution of sequences heterozygous at two or more loci, typically not required for diplotypic MLST typing schemes. However, haplotypes can be derived from current targets reconstructed by the program PHASE [51], or more recently fastPHASE (http://depts.washington.edu/uwc4c/express-licenses/assets/fastphase/), by cloning, or by allelic specific PCR [52]. In a sexually reproducing population the frequencies of genotypes (defined by haplotypes) should be in Hardy-Weinberg equilibrium. New software to analyse haplotypic data include those incorporating Bayesian methodologies; Structure [53] and Beast [54] reconstruct phylogenies with epidemiologically and evolutionary informative results.

### Loss of heterozygosity

LOH observed in 4 gene fragments affecting the hybrid lineages TcV and TcIV has potentially significant consequences for MLST and lineage assignment. For example, *TcMPX* hybrid isolates (TcV) would be incorrectly assigned if judgment was based on a single locus due to LOH (Figure 1). There was no continuity of allelic loss across different genes for individual isolates, linked genes (chromosome 36) were homozygous in *Met-III* but heterozygous in *TcAPX* indicating local rather than large scale events indicative of LOH seen in *C. albicans* [55]. Such apparent random loss is consistent with gene conversion. However, within *TcMPX* all TcV isolates (with the exception of PAH179) possessed only a TcII like allele suggesting clonal expansion subsequent to this LOH event. Discriminating between recombination, gene conversion, duplication or mitotic recombination requires analysis at the haplotypic level and should be possible by MLST, particularly in light of the considerable heterozgosity found in a *T. cruzi* (in particular DTUs TcI, TcIII, and TcIV). Use of a larger number of sympatric isolates and gene targets evenly distributed along those chromosomes where LOH has been previously identified (chromosomes 6, 36, 29, 22) would be a suitable approach, and one that has been exploited in *C. albicans* [55]. LOH in *C. albicans* [56], [57], [58] is one mechanism generating minor genetic changes which over time enable adaptation to new microenvironments [59], [60], [61], [62]. Such microvariation has been demonstrated by passage of *C. albicans in vitro*. Prolonged passage of MLST characterised *T.cruzi* isolates under a range of different environmental stresses could be applied to detect genetic basis for fitness. In the current panel 40% of the SNPs were non synonymous and resulted in amino acid changes. Some of these were nontrivial (e.g., acidic to basic side chains, aliphatic to aromatic side chains) which would be expected to result in significant changes in higher and secondary peptide structures and inter and intralineage catalytic/phenotypic differences.

Taken together we demonstrate that diploid sequence typing is a powerful and practical method to describe the extent of diversity in *T. cruzi* that can be integrated into population and evolutionary studies, particularly if the current protocol can be applied to an MLST database for *T. cruzi*. We propose that four genes (*Met-III*, *RB19*, *TcGPXII* and *DHFR-TS*) be used for the characterisation of isolates to DTU level. Additionally we propose that six gene fragments (*Met-II*, *Met-III*, *RB19*, *TcMPX*, *DHFR-TS* and *TR*) form the basis for a formalised MLST diplotyping scheme for further high resolution studies on the basis that DST diversity differentiated 38 out of 39 isolates. The proposed panel does not exclude the addition or replacement of new gene targets to further optimise diversity studies. eBURST analysis predicts that DST 29 isolated from *T. infestans* in Boliva and Chile is the genetic founder of all other TcV isolates in the reference panel (although isolate numbers were small), phylogenies also support a cladistic subdivision between North and South American TcIV isolates. SNP data revealed that for three genes (*LYT1*, *TcGPXII* and *TcMPX)* putative donor homozygous SNP profiles and the corresponding heterozygous profiles, were present within DTUs TcI and TcIII for single isolates. Incongruent topologies and the intra and inter DTU level is not consistent with a model of strict clonality. The high levels of heterozygosity detected in the gene targets offers the potential for a future deeper level of analysis of population genetics based on haplotypes.

## Supporting Information

Figure S1 Concatenation and lineage assignment. Unrooted neighbor-joining diplotypic tree showing *p*-distance for 9 concatenated gene fragments (A). Concatenation of *LYT1* and *DHFR-TS* discriminate between DTUs V and VI (inset). Concatenated diplotypic tree using a reduced panel of 4 gene fragments (B). Concatenation of *Met-III*, *RB19*, and *TcGPXII* assign isolates to DTUs TcI-TcIV. *DHFR-TS* differentiates DTUs V and VI (B inset).
(0.41 MB DOC)
