# Supplementary figures and images for: Multilocus Sequence Typing (MLST) for Lineage Assignment and High Resolution Diversity Studies in Trypanosoma cruzi
Source: PLoS Negl Trop Dis. 2011 Jun 21;5(6):e1049. doi: 10.1371/journal.pntd.0001049 (PMC3119646; doi:10.1371/journal.pntd.0001049)

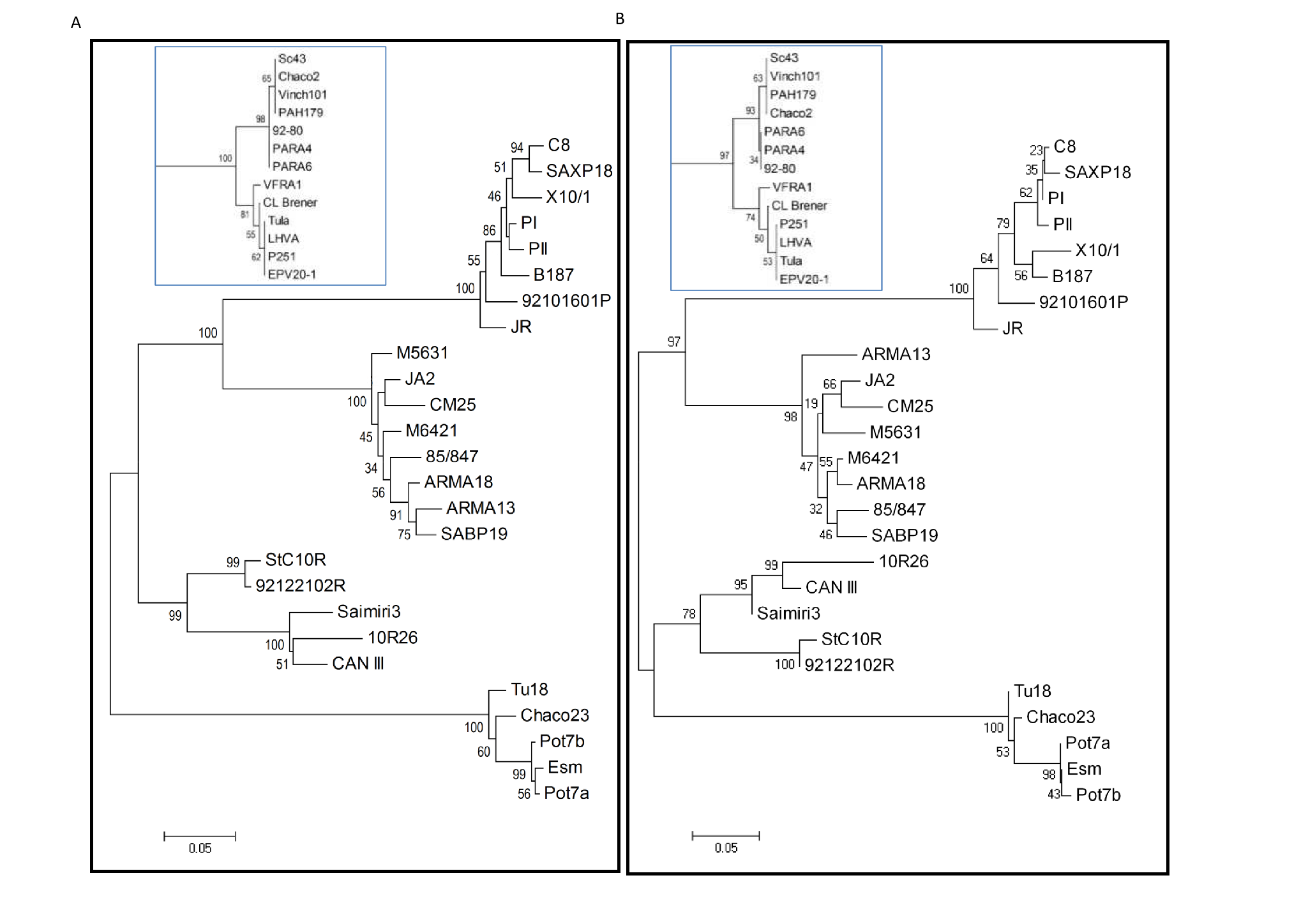

Supplement: Figure S1 — Concatenation and lineage assignment. Unrooted neighbor-joining diplotypic tree showing p-distance for 9 concatenated gene fragments (A). Concatenation of LYT1 and DHFR-TS discriminate between DTUs V and VI (inset). Concatenated diplotypic tree using a reduced panel of 4 gene fragments (B). Concatenation of Met-III, RB19, and TcGPXII assign isolates to DTUs TcI-TcIV. DHFR-TS differentiates DTUs V and VI (B inset). (0.41 MB DOC) [file pntd.0001049.s001.doc]
